# Prevalence, Risk Factors, and Perinatal Outcomes of Velamentous Umbilical Cord Insertion in Twin Pregnancies: A Single-Center Retrospective Study

**DOI:** 10.3390/jcm13051396

**Published:** 2024-02-28

**Authors:** Ayaho Somiya, Hiroyuki Tsuda, Eri Tsugeno, Yumi Nakamura, Masafumi Kuroyanagi, Hajime Araki, Yoshiki Masahashi, Miho Suzuki, Nobuhiko Fukuhara, Yumiko Ito, Atsuko Tezuka, Tomoko Ando, Kimio Mizuno

**Affiliations:** Department of Obstetrics and Gynecology, Japanese Red Cross Aichi Medical Center Nagoya Daiichi Hospital, Nagoya 453-85511, Japan; f.ayaho.med@gmail.com (A.S.); eri.uchiyama.56@gmail.com (E.T.); yuim0201@gmail.com (Y.N.); ma.sakuro1121@gmail.com (M.K.); hajiwing@icloud.com (H.A.); yoshiki070307030703@gmail.com (Y.M.); miho418miho@gmail.com (M.S.); m06087nf@jichi.ac.jp (N.F.); yum.ito.jrc@gmail.com (Y.I.); atezuka229@gmail.com (A.T.); ando-tm@nagoya-1st.jrc.or.jp (T.A.); kimizuno@nagoya-1st.jrc.or.jp (K.M.)

**Keywords:** chorionicity, fetal growth restriction, preterm birth, twin pregnancy, velamentous cord insertion

## Abstract

Background: The effect of velamentous cord insertion (VCI) on perinatal outcomes in twin pregnancies is unclear due to conflicting findings. This retrospective study aimed to examine VCI prevalence and related risk factors in twin pregnancies and its association with adverse perinatal outcomes. Methods: Women with twin pregnancies who delivered between January 2012 and December 2021 in a single tertiary hospital were included. The participants were divided into dichorionic (DC) and monochorionic diamniotic (MCDA) groups, and their maternal and fetal characteristics and VCI rates were compared. Logistic regression models were used to identify risk factors for VCI and VCI-related perinatal outcomes. Results: Among the 694 twin pregnancies included in this study, the VCI rate was significantly higher in MCDA than in DC twins. Body mass index and MCDA twins were significant risk factors for VCI, whereas assisted reproductive technology pregnancy was a significant protective factor against VCI. In DC twins, VCI did not affect perinatal outcomes. In MCDA twins, VCI was a significant risk factor for fetal growth restriction, twin-to-twin transfusion syndrome, and preterm birth at <36 weeks. Conclusions: VCI was a prominent risk factor for adverse perinatal outcomes only in MCDA twins. Antenatal sonographic assessment of the umbilical cord insertion site would be beneficial.

## 1. Introduction

Velamentous cord insertion (VCI) is defined as the abnormal insertion of the umbilical vessels into the fetal membranes without Wharton’s jelly before entering the placenta. The membranous vessels are at risk of rupture, kinking, and compression, which increases the risk of adverse pregnancy outcomes [1,2]. The reported incidence of VCI is approximately 0.1–1.8% in all pregnancies [3] and as high as 40% in twin pregnancies [3,4,5,6]. In a study examining 1498 twin placentas after delivery, the prevalence of VCI in dichorionic (DC) and monochorionic diamniotic (MCDA) twin pregnancies without and with twin-to-twin transfusion syndrome (TTTS) was 7.6, 34.7, and 36.1, respectively [7]. A recent study reported a VCI prevalence in DC and MCDA twins of 5.8% and 7.8%, respectively [8]. 

VCI contributes to adverse maternal and perinatal outcomes [1,2,3,4]. However, findings are controversial regarding its effect on perinatal outcomes in twin pregnancies. Costa-Castro et al. found that VCI increased the risk of adverse outcomes (such as severe birthweight discordance and/or small for gestational age) in MCDA twins but not in DC twins [7]. Furthermore, VCI was reported to be a risk factor for selective fetal growth restriction (sFGR), TTTS, and birthweight discordance in MCDA twins [4,9,10]. In contrast, other studies concluded that the adverse complications in pregnancies with VCI were probably due to selection bias, noting that these specific adverse outcomes in MCDA twins were more likely the result of vascular complications due to placental sharing rather than VCI [8,11]. Recently, we reported that VCI and marginal cord insertion in the second twin were significant risk factors for umbilical cord prolapse during vaginal delivery [12].

This study aimed to examine VCI prevalence and related risk factors in twin pregnancies and to evaluate its effect on perinatal outcomes according to chorionicity. We hypothesized that VCI would be a significant risk factor for perinatal complications such as FGR and TTTS in MCDA twins, but not in DC twins.

## 2. Materials and Methods

### 2.1. Study Design and Population

This was a single-center retrospective study that included women with twin pregnancies who delivered at our institution between January 2012 and December 2021. The exclusion criteria were as follows: (1) monochorionic monoamniotic twin pregnancies; (2) twin pregnancies with unknown chorionicity; (3) missing information regarding the umbilical cord insertion site; and (4) intrauterine fetal death. 

This study conformed with the principles outlined in the Declaration of Helsinki of 1964. The study was approved by the Ethics Committee of the Japanese Red Cross Aichi Medical Center Nagoya Daiichi Hospital, Nagoya, Japan (approval number: 2023-162). Informed consent was not required for this study since we used existing anonymous clinical data.

### 2.2. Pregnancy Management 

After confirming twin pregnancy, chorionicity and amnionicity were determined via ultrasound in the first trimester. In brief, the presence of the “twin peak” or “lambda” sign at the inter-twin membrane indicated DC twins, while the presence of the “T” sign indicated MCDA twins. Pregnancy check-ups were performed every 2 weeks after gestational week 16 for MC twins and after gestational week 22 for DC twins. At each check-up, we assessed the fetal position, amniotic fluid volume, and fetal growth. Additionally, umbilical artery Doppler was performed for all MCDA twins and for DC twins with FGR and/or oligohydramnios. Our hospital policy is to deliver DC twins at gestational week 37 and MCDA twins at gestational week 36. After delivery, the length, insertion site, number of vessels, and gross abnormalities (e.g., knots) of the umbilical cord were confirmed by gross examination.

### 2.3. Data Collection and Definitions 

Data on maternal and fetal characteristics were extracted from the patients’ medical records. The method of conception was categorized as spontaneous, induction, or assisted reproductive technology (ART). In Japan, the number of embryos transferred in ART pregnancies follows the guidelines of the Japan Society for Reproductive Medicine. The number of embryos transferred (one or two) depends on factors such as age and history of fertility treatment. Preeclampsia was defined as the presence of gestational hypertension (blood pressure >140/90 mmHg after 20 weeks of gestation), proteinuria (>300 mg per day), and/or findings of end-organ dysfunction during pregnancy [13]. FGR was defined as a fetal body weight of less than −1.5 SD for the gestational age [14]. TTTS was defined as discordant amniotic fluid volumes (maximal vertical pocket less than 2 cm and more than 8 cm in each amniotic sac at the same time) in MCDA twins [15]. Neonatal weight was defined as the weight at birth.

### 2.4. Statistical Analyses

Extracted data were entered into a computerized spreadsheet (Excel, Microsoft Japan Co., Ltd., Tokyo, Japan). EZR software (version 1.38, Saitama, Japan) was used for all data analyses. For data analysis, patients were divided into two groups: DC and MCDA. After assessing data normality using the Shapiro–Wilk test, the Mann–Whitney *U* test or Student’s *t*-test was used to compare continuous variables between the two groups, as appropriate. Continuous variables included maternal age, gestational age at delivery, body mass index (BMI), and birth weight. The chi-squared test was performed to compare the following categorical variables: chorionicity, nulliparity, ART, male infant, preterm birth, diabetes mellitus, preeclampsia, FGR, and VCI. Logistic regression models were employed to identify risk factors for VCI (maternal age, BMI, nulliparity, ART, and monochorionic twin) and the prognostic value of VCI for perinatal outcomes (maternal age, BMI, nulliparity, ART, diabetes mellitus, preeclampsia, and FGR). For each variable, the adjusted odds ratio (aOR) and 95% confidence interval (CI) were estimated. *p*-values less than 0.05 were considered to indicate statistical significance.

### 2.5. Patient and Public Involvement 

Neither patients nor the public were involved in the design, conduct, reporting, or dissemination of this research.

## 3. Results

### 3.1. Participant Selection Process

Among the 716 women with twin pregnancies identified, 22 were excluded from the study (1 with monochorionic monoamniotic twin pregnancy, 4 with unknown chorionicity, and 17 with missing information on the umbilical cord insertion site). Finally, 694 women with twin pregnancies (including 451 DC and 243 MCDA pregnancies) were included (Figure S1).

### 3.2. Maternal Characteristics and Delivery Outcomes

The maternal and fetal characteristics are shown in Table 1. Pre-pregnancy maternal BMI and ART rate were significantly higher in DC than in MCDA twin pregnancies. Gestational age at delivery and neonatal body weight were significantly higher in DC than in MCDA twins. However, the rates of preterm birth at <36 weeks and FGR were higher in MCDA than in DC twins. Additionally, the VCI rate was significantly higher in MCDA than in DC twins (9.7% vs. 4.4%, *p* < 0.001).

### 3.3. Maternal Characteristics in VCI Cases

In DC twins, the rate of ART pregnancies was significantly lower among VCI than among non-VCI cases, and VCI was a significant risk factor for preterm birth at <36 weeks (Table S1). In MCDA twins, VCI was a significant risk factor for preterm birth at <36 weeks and TTTS (Table S2). No differences were observed in the incidence of VCI by mode of conception.

### 3.4. Risk Factors for VCI

In the multivariate analyses for risk factors for VCI, maternal BMI and MCDA pregnancy were significant risk factors for VCI (aOR, 1.09; 95% CI: 1.02–1.16; *p* = 0.009 and aOR, 2.34; 95% CI: 1.42–3.84; *p* < 0.001, respectively), while ART pregnancy was a significant protective factor against VCI (aOR, 0.452; 95% CI: 0.214–0.954; *p* = 0.037) (Table 2). 

### 3.5. VCI and Perinatal Outcomes

The multivariate analysis results for the prognostic role of VCI for perinatal outcomes are shown in Table 3. In DC twins, no significant association was observed between VCI and perinatal outcomes (FGR and preterm birth at <36 weeks). In contrast, in MCDA twins, VCI was a significant risk factor for the following adverse perinatal outcomes: FGR (aOR, 3.01; 95% CI: 1.36–6.67; *p* = 0.007), TTTS (aOR, 5.22; 95% CI: 2.04–13.4; *p* < 0.001), and preterm birth at <36 weeks (aOR, 3.25; 95% CI: 1.54–6.85; *p* = 0.002). Furthermore, no significant association was observed between VCI and preterm birth at <34 weeks and <28 weeks in DC twins; however, VCI was a significant risk factor for preterm birth at <34 weeks (aOR, 3.00; 95% CI: 1.47–6.11; *p* = 0.002) and <28 weeks (aOR, 3.92; 95% CI: 1.34–11.5; *p* = 0.013) in MCDA twins.

## 4. Discussion

### 4.1. Main Findings

In the present study, the VCI rate was 9.7% in MCDA twins and 4.4% in DC twins, with it being significantly higher in MCDA twins. Maternal BMI and MCDA pregnancy were identified as significant risk factors for VCI, while ART pregnancy was a significant protective factor. VCI was not associated with perinatal outcomes in DC twins, whereas in MCDA twins, it was associated with FGR, TTTS, and preterm birth at <36, 34, and 28 weeks. 

### 4.2. Interpretation

The reported prevalence of VCI in twin pregnancies ranges from 1.6% to 40% [3,4,5,6]. The VCI rates in the current study were similar to those of 5.8% in DC twins and 7.8% in MCDA twins reported by Lee et al. [8]. Furthermore, the VCI rate was significantly higher in MCDA than in DC twins, which aligns with the findings of Costa-Castro et al. [7]. Contrarily, other studies have reported similar VCI rates in DC and MCDA twins [8,16]. Nonetheless, the VCI rate is considered to be higher in twin pregnancies than in singleton pregnancies.

Regarding the impact of VCI on perinatal outcomes in twin pregnancies, the reported findings are contradictory. Some studies have found that VCI is a risk factor for adverse perinatal outcomes in MCDA twins [4,7,9,10,17], and that perinatal mortality is notably higher in VCI than in non-VCI MCDA twins (30% vs. 10.5%) [18]. In contrast, in other studies, VCI in twin pregnancies was not associated with adverse perinatal outcomes in either in MCDA or in DC twins [8]. In the current study, we observed no significant association between VCI and perinatal outcomes in DC twins; however, VCI was associated with FGR, TTTS, and preterm birth at <36, 34, and 28 weeks in MCDA twins. These findings suggest that we need to be vigilant about VCI in MCDA twins, which necessitates specialized management, including frequent check-ups of cervical length, fetal growth, amniotic fluid volume, and fetal blood flow measurements in such cases. However, this study evaluated data on only prenatal outcomes not perinatal outcomes. It should therefore be noted that in terms of neonatal outcomes, an association with VCI was not shown in this study and thus, cannot be compared with the previous reports mentioned above.

In our study, MCDA pregnancy was identified as a significant risk factor for VCI (aOR, 2.34), whereas ART pregnancy was a significant protective factor against VCI (aOR, 0.452). In previous studies, VCI was reported to have a varying degree of association with in vitro fertilization (IVF) compared with natural pregnancy [3,4,16,19]. Furthermore, in a systematic review, ART pregnancy was associated with a higher rate of VCI compared with natural pregnancy, and the VCI rate was similar among the different ART methods (blastocyst vs. cleavage-stage transfer and frozen vs. fresh embryo transfer) [20]. However, these studies included singleton pregnancies only [3,19], or both singleton and multiple pregnancies [4,20], and none included only multiple pregnancies. Delbaere et al. reported an increasing incidence of VCI according to the invasiveness of the reproductive technique in dizygotic twins: 3.6% in natural conception, 5% in artificial induction of ovulation, 7.4% in IVF, and 10.4% in intracytoplasmic sperm injection [21]. Although our results differ from those of previous studies, few studies on ART pregnancies and VCI risk have been conducted among twin pregnancies only. 

**Figure 1:**
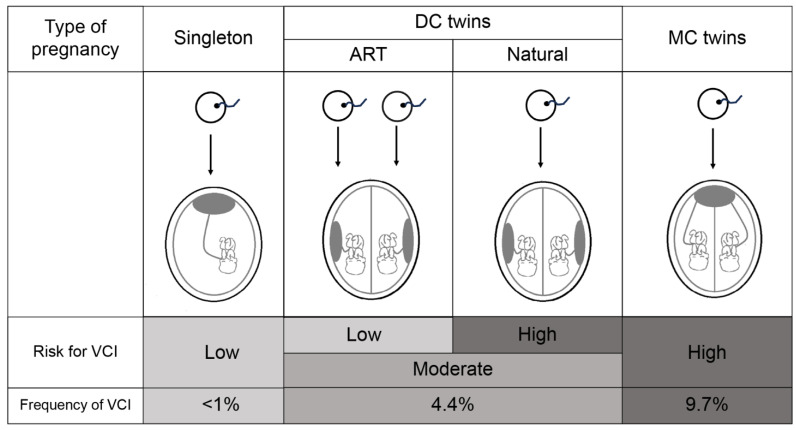
Our speculation with regard to the results that ART pregnancies reduce the risk of VCI in twin pregnancy. VCI, velamentous cord insertion; DC, dichorionic; MC, monochorionic; ART, assisted reproductive technology.

With regard to our finding that ART pregnancies reduced the risk of VCI, we speculate the reason to be as follows. The fact that singleton pregnancies have the lowest and MCDA pregnancies have the highest risk of VCI suggests that the reason may be the splitting of monozygotic twins after fertilization. In DC twins, most ART pregnancies involve dizygotic and no splitting processes, and therefore follow a similar process to that in singleton pregnancies. Contrarily, naturally conceived DC twins have a higher risk of VCI than those conceived by ART because most of them undergo the same process of splitting after fertilization, similar to MCDA twins (Figure 1). If ART pregnancy reduces the risk of VCI, particularly considering that ART pregnancies are more common in DC twins [22], this would be positive information for the patient. Further analysis of the data on the number of embryos transferred in ART pregnancies is needed to support this hypothesis. Those data were not available for this study and need to be verified in the future. We recognize that the results of the present analysis differ in many areas from previous reports and that there is no consensus. Furthermore, the association cannot be necessarily causal. However, our findings are novel, with few previous reports on twin pregnancies. Therefore, we believe that these results should be validated in the future.

In the present study, data on the umbilical cord insertion site was obtained by gross examination after delivery. As the study was retrospective, data on prenatal umbilical cord insertion sites obtained through ultrasound examination were not complete. The overall accuracy of screening for VCI using transabdominal sonography (TAS) is considerably high [16]. Second-trimester TAS has been reported to have consistently high specificity (>99.8%) [23,24,25]. In the largest and highest-quality study, sensitivity was reported to be 62.5%, specificity 100%, positive predictive value 83%, negative predictive value 100%, and accuracy 99.8% [25]. Although these data are from singleton pregnancies, we believe that the same high accuracy can be achieved in twin pregnancies. A well-designed prospective study with a larger cohort of women could examine this issue further. Accurate diagnosis of the umbilical cord insertion site via second-trimester TAS in twin pregnancies will require more careful observation for preterm birth, FGR, and TTTS, particularly in MCDA twins, leading to better pregnancy management.

### 4.3. Strengths and Limitations

The main strength of this study is that it was conducted at a single center and included a relatively large sample size (694 twin cases). Due to the single-center design, pregnancy management and treatment policies were homogeneous, and a certain quality of practice was maintained. Moreover, the institution complies with the Guideline for Obstetrical Practice in Japan and provides a standard of care. Another strength is the accuracy of the diagnosis of VCI, as it was made by macroscopic examination of the placenta after delivery.

The main limitation of this study is its retrospective nature, which limits the reliability of the results due to selection biases. Furthermore, VCI is associated with an increased risk of hemorrhage in the third stage of labor and the need for manual removal of the placenta [26]. In addition, VCI can sometimes coincide with vasa previa [16,27]. However, we did not have data regarding these adverse outcomes. In this study, there was no data on sFGR, which applies only to MCDA twins. We also did not examine between-twin differences in birthweight. These data are also very important and require further study. Finally, the mechanism underlying the low rate of VCI in ART pregnancies remains unclear and should be examined in future studies.

The optimal obstetric strategy is for VCI to be diagnosed not after delivery but during pregnancy (optimally in the second trimester), which would make it possible to predict severe complications in the third trimester and during delivery, especially for MCDA twins. In the future, it is important to confirm that antenatal TAS can be used to diagnose VCI with a high degree of accuracy, and if so, from a clinical perspective, cases with antenatal diagnosis of VCI should be managed carefully to ensure better pregnancy outcomes for the mother and child. 

## 5. Conclusions

The VCI rate in our study was significantly higher in MCDA than in DC twins. MCDA pregnancy was a significant risk factor for VCI, while ART pregnancy was a significant protective factor. VCI was a significant risk factor for adverse perinatal outcomes in MCDA twins, but not in DC twins. A notable finding is that ART pregnancies reduced the risk of VCI, which has rarely been reported. Further research is warranted to clarify the underlying mechanisms. We believe that the findings of this study will provide valuable and novel information for the future management of twin pregnancies.

## Figures and Tables

**Table 1:** Baseline maternal and fetal characteristics.

	Dichorionic Twins (*n* = 451)	Monochorionic Twins (*n* = 243)	*p*-Value
Maternal age ^a^ (years)	33 (19–53)	32 (17–44)	0.117
Pre-pregnancy BMI ^a^ (kg/m^2^)	20.5 (13.8–39.4)	20.1 (15.6–33.9)	0.030
Nulliparous	125/451 (27.7%)	63/243 (25.9%)	0.388
ART	149/451 (33.0%)	21/243 (8.6%)	<0.001
GA at delivery ^a^ (weeks)	36.7 (22.9–39.3)	36.0 (24–39.4)	<0.001
Neonatal body weight (g)	2205 (±490)	1973 (±602)	<0.001
Male infant	473/902 (52.4%)	276/486 (56.8%)	0.286
Preterm birth at <36 week	142/451 (31.5%)	111/243 (45.7%)	<0.001
Preeclampsia	69/451 (15.3%)	29/243 (11.9%)	0.254
Diabetes mellitus	16/451 (3.5%)	6/243 (2.5%)	0.288
Fetal growth restriction	216/902 (23.9%)	163/486 (33.5%)	<0.001
Velamentous cord insertion	40/902 (4.4%)	47/486 (9.7%)	<0.001
Vaginal delivery	195/451 (43.2%)	95/243 (39.1%)	0.478

^a^ Median (range); BMI, body mass index; ART, assisted reproductive technology; GA, gestational age.

**Table 2:** Multivariate logistic regression analysis for risk factors for velamentous cord insertion.

	Adjusted OR	95% CI	*p*-Value
Maternal age	0.981	0.935–1.03	0.430
Pre-pregnancy BMI	1.090	1.02–1.16	0.009
Nulliparity	0.906	0.526–1.56	0.723
ART	0.452	0.214–0.954	0.037
Monochorionic twins	2.340	1.42–3.84	<0.001

BMI, body mass index; ART, assisted reproductive technology; OR, odds ratio; CI, confidence interval.

**Table 3:** Multivariate logistic regression analysis of the risk of adverse perinatal outcomes with velamentous cord insertion.

	Adjusted OR	95% CI	*p*-Value
Dichorionic twins			
Fetal growth restriction ^a^	1.160	0.254–5.29	0.850
Preterm birth at <36 weeks ^b^	1.970	0.956–4.05	0.066
Monochorionic twins			
Fetal growth restriction ^a^	3.010	1.36–6.67	0.007
Twin-to-twin transfusion syndrome ^c^	5.220	2.04–13.4	<0.001
Preterm birth at <36 weeks ^b^	3.250	1.54–6.85	0.002

OR, odds ratio; CI, confidence interval. ^a^ Covariates: maternal age, pre-pregnancy body mass index, assisted reproductive technology, preeclampsia, and diabetes mellitus; ^b^ covariates: maternal age, pre-pregnancy body mass index, nulliparity, assisted reproductive technology, preeclampsia, diabetes mellitus, and fetal growth restriction; ^c^ covariates: maternal age, pre-pregnancy body mass index, nulliparity, and assisted reproductive technology.

## Data Availability

The data that support the findings of this study are available from the corresponding author upon reasonable request (email: hirotty7099@yahoo.co.jp).

## Supplementary Materials

The following supporting information can be downloaded at: https://www.mdpi.com/article/10.3390/jcm13051396/s1. Table S1: Maternal characteristics in dichorionic twins by cord insertion type. Table S2: Maternal characteristics in monochorionic twins by cord insertion type. Figure S1: Study flowchart.
