# Supplementary material for: Prevalence, Risk Factors, and Perinatal Outcomes of Velamentous Umbilical Cord Insertion in Twin Pregnancies: A Single-Center Retrospective Study
Source: J Clin Med. 2024 Feb 28;13(5):1396. doi: 10.3390/jcm13051396 (PMC10932138; doi:10.3390/jcm13051396)
Supplement: Supplementary file 1 [file jcm-13-01396-s001.zip › jcm-2885774-supplementary-tab.pdf]

Supplementary Table S1. Maternal characteristics in dichorionic twins by cord insertion type.

	Velamentous (n=35)	Normal (n=416)	*P*-value
Preterm birth at <36 weeks	17/35 (48.6%)	125/416 (30.0%)	0.036
Preterm PROM	5/35 (14.3%)	30/416 (7.2%)	0.176
Preeclampsia	8/35 (22.9%)	61/416 (14.7%)	0.219
Diabetes mellitus	0/35 (0%)	16/416 (3.8%)	0.625
Mode of conception			0.026
Natural	12/35 (34.3%)	156/416 (37.5%)	
Induction	17/35 (48.6%)	117/416 (28.1%)	
ART	6/35 (17.1%)	143/416 (34.4%)	

PROM, premature rupture of membrane; ART, assisted reproductive technology.

Supplementary Table S2. Maternal characteristics in monochorionic twins by cord insertion type.

	Velamentous (n=44)	Normal (n=199)	*P*-value
Preterm birth at <36 weeks	31/44 (70.5%)	80/199 (40.2%)	<0.001
Preterm PROM	3/44 (6.8%)	16/199 (8.0%)	1
Preeclampsia	3/44 (6.8%)	26/199 (13.1%)	0.312
Diabetes mellitus	2/44 (4.5%)	5/199 (2.5%)	0.613
Mode of conception			0.695
Natural	35/44 (79.5%)	143/199 (71.9%)	
Induction	6/44 (13.6%)	38/199 (19.1%)	
ART	3/44 (6.8%)	18/199 (9.0%)	
TTTS	11/44 (25%)	12/199 (6.0%)	<0.001

PROM, premature rupture of membrane; ART, assisted reproductive technology; TTTS, twin-to-twin transfusion syndrome.
