# Supplementary figures and images for: Prevalence, Risk Factors, and Perinatal Outcomes of Velamentous Umbilical Cord Insertion in Twin Pregnancies: A Single-Center Retrospective Study
Source: J Clin Med. 2024 Feb 28;13(5):1396. doi: 10.3390/jcm13051396 (PMC10932138; doi:10.3390/jcm13051396)

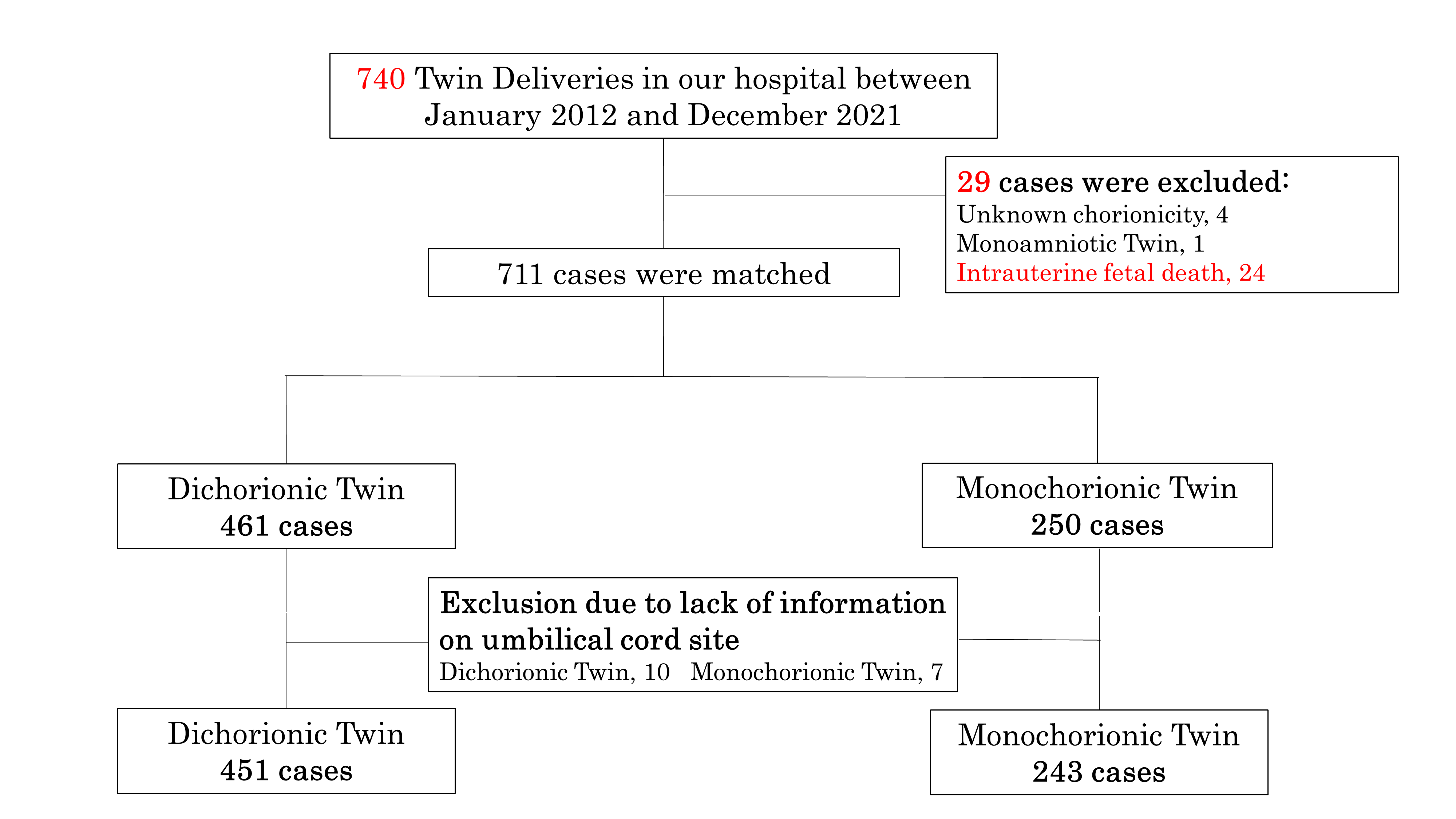

Supplement: Supplementary file 1 [file jcm-13-01396-s001.zip › jcm-2885774-FigureS1.tif]
